# Hydrolyzed collagen intake increases bone mass of growing rats trained with running exercise

**DOI:** 10.1186/1550-2783-10-35

**Published:** 2013-08-06

**Authors:** Satoko Takeda, Jong-Hoon Park, Eriko Kawashima, Ikuko Ezawa, Naomi Omi

**Affiliations:** 1 Graduate School of Comprehensive Human Sciences, University of Tsukuba, Ibaraki, Japan; 2 Center for Disease Biology and Integrative Medicine, University of Tokyo, Tokyo, Japan; 3 Department of Physical Education, Konkuk University, Seoul, South Korea; 4 Yokohama Research Center, JNC Corporation, Yokohama, Japan; 5 Japan Women’s University, Tokyo, Japan; 6 Faculty of Health and Sport Sciences, University of Tsukuba, Ibaraki, Japan; 7 Institute of Health and Sports Sciences, Graduate School of Comprehensive Human Sciences, University of Tsukuba, Tsukuba, Ibaraki 305-8574, Japan

**Keywords:** Hydrolyzed collagen peptides, Bone mass, Bone strength, Physical exercise, Growing phase, Rats

## Abstract

**Background:**

Some studies have shown that dietary hydrolyzed collagen peptides (HC) effectively prevent age-related bone loss. However, it is not known whether the intake of HC also has positive effect on bone mass or strength when combined with exercise during growth phase.

**Methods:**

We examined the effects of 11 weeks of HC intake and running exercise on bone mass and strength in growing rats. Rats were randomized into four groups, the 20% casein group (Casein20), the 40% casein group (Casein40), the 20% HC group (HC20), and the 40% HC group (HC40). Each group was further divided into exercise groups (Casein20 + Ex, Casein40 + Ex, HC20 + Ex, HC40 + Ex) and non-exercise group (Casein20, Casein40, HC20, HC40). In the HC intake groups, 30% of casein protein was replaced with HC. Exercise group rats were trained 6 days per week on a treadmill (25–30 m/min, 60 min) for 60 days. After being sacrificed, their bone mineral content (BMC) and bone strength were evaluated.

**Results:**

Exercise and dietary HC effects were observed in the adjusted BMC of lumbar spine and tibia among the 20% protein groups (p < 0.001 for exercise; p < 0.05 for dietary HC, respectively). These effects were also noted in the adjusted wet weight and dry weight of femur among the 20% protein groups (p < 0.001, p < 0.01 for exercise; p < 0.01, p < 0.001 for dietary HC, respectively). On the other hand, in adjusted bone breaking force and energy, dietary HC effect was not significant. Among the 40% protein groups, similar results were obtained in the adjusted BMC, femoral weight, bone breaking force, and energy. There were no differences between the 20% protein groups and the 40% protein groups.

**Conclusions:**

The present study demonstrated that moderate HC intake (where the diet contains 20% protein, of which 30% is HC) increased bone mass during growth period and further promoted the effect of running exercise. On the other hand, a higher HC intake (where the diet contains 40% protein, of which 30% is HC) had no more beneficial effect on bone mass than the moderate HC intake.

## Background

It is generally well accepted that physiologically mechanical loading, e.g., physical activity or exercise, plays important roles in having higher bone mass during growth period [1]. In addition, nutritional factors such as protein are essential for increasing bone formation [2]. Thus, for achieving peak bone mass during growing phase, not only mechanical loading but also sustaining adequate protein intake may be of significance. In particular, although young athletes involved in physical training have high protein intakes [3], the effects of protein intake and physical exercise on growing bone have not been well understood.

Type I collagen is the major structural protein, being the main extra cellular matrix protein for calcification. It is distributed throughout the whole body accounting for 25% of total body protein and for 80% of total conjunctive tissue in humans [4]. The synthesis of type I collagen also plays a role in further promoting osteoblast differentiation [5,6]. Collagen peptides, the enzymatic degradation products of collagens, have recently been shown to have several biological activities, and have been used as preservatives [7-9]. The intake of hydrolyzed collagen peptides (HC) in animal models has been shown to improve bone mineral density (BMD) and bone mineral content (BMC) [10], and to increase the quantity of type I collagen in bone matrix of ovariectomized (OVX) rats [11]. Moreover, in patients with osteoporosis, oral intake of HC in addition to injection of calcitonin had a stronger inhibitory effect on bone resorption than the injection of calcitonin alone [12]. These results suggest that dietary collagen peptides would effectively prevent age-related bone loss. However, it has not been demonstrated whether the intake of HC also has positive effect on bone mass or strength in growing bone.

Some studies have investigated the effects of the intake level of protein on bone mass. Protein deficiency could decrease the secretion of insulin-like growth factor 1 (IGF-1) [13], which may prevent normal growth of bone mass. Recently, we also demonstrated that a low protein intake suppressed the acquisition of bone mass and the increase of bone strength during growth period [14]. Conversely, a high protein intake results in higher urinary calcium (Ca) excretion, which may lead to accelerated bone resorption [15]. Similarly, we demonstrated that a high protein intake suppressed the increase of bone strength during growth period in which treadmill running was performed [14]. However, these studies used only casein protein as a protein source of the diet; it is not known whether HC intake included in a high protein diet has positive effect on bone mass or strength when combined with running exercise during growth phase.

Accordingly, the aim of this study is to investigate 1) the effect of HC intake alone and HC intake combined with treadmill running exercise on bone mass and strength in growing rats, 2) whether the intake of a high protein diet containing HC has a positive effect on bone mass and strength of growing rats trained with running exercise.

## Methods

### Experimental animals and protocol

Fifty-nine male Wistar rats, 5 weeks of age were obtained from CLEA Japan, Inc (Tokyo, Japan). Rats were randomized into four groups, the 20% casein group (Casein20), the 40% casein group (Casein40), the 20% HC group (HC20), and the 40% HC group (HC40). Each group was further divided into exercise groups (Casein20 + Ex, Casein40 + Ex, HC20 + Ex, HC40 + Ex) and non-exercise groups (Casein20, Casein40, HC20, HC40) (n = 7 or 8 each). The experimental period was 11 weeks. The animals were individually housed at 23 ± 1°C and humidity of 50 ± 5% on an inverted 12/12 h light/dark cycle. All animals received food and water *ad libitum*. Body weight and food intake were measured at 48 h intervals throughout the experimental period. All experimental protocols in the present study were approved by the Committee on Animal Research at the University of Tsukuba.

### Experimental diets

Each group received one of two levels of protein for its diet, 20% or 40% to total diet weight. Since the recommended dietary percentage of protein for growing animals is 17.9% [16], we used 20% protein as a moderate protein intake and 40% protein as a high protein intake. In the HC intake groups, 30% of casein protein was replaced with HC. Enzymatic HC was provided by JNC Corporation (Yokohama, Japan). The HC was of marine fish origin with a molecular weight of about 1 kDa. It was prepared with extraction by the enzymatic degradation. Then the extracted product was concentrated and dried. The product is powder with little taste and odor. All diets were controlled at 0.6% Calcium (Ca) and 0.6% Phosphate (P). These diet compositions are described in Table 1.

**Table 1:** Composition of experimental diets

**Constituents**	**20% Protein**		**40% Protein**	
	**collagen(-)**	**collagen(+)**	**collagen(-)**	**collagen(+)**
	**(0.6% Ca, 0.6% P)**	**(0.6% Ca, 0.6% P)**	**(0.6% Ca, 0.6% P)**	**(0.6% Ca, 0.6% P)**
Glucose monohydrate	60.4	60.3	40.8	40.6
Casein (Vitamin free)	20.0	14.0	40.0	28.0
Hydrolyzed collagen	_	6.0	_	12.0
Cystine	0.2	0.2	0.2	0.2
Cottonseed oil	10.0	10.0	10.0	10.0
CaCO_3_	1.4879	1.4777	1.4774	1.4734
KH_2_PO_4_	1.1424	0.9667	0.9666	1.0636
K_2_HPO_4_	1.4621	1.2373	1.2372	1.3613
Roughage	3.0	3.0	3.0	3.0
Choline chloride	0.2	0.2	0.2	0.2
Water soluble Vitamin mixture^a)^	0.1	0.1	0.1	0.1
Oil soluble Vitamin mixture	b)	b)	b)	b)
Ca P free salt mixture^c)^	2.0	2.0	2.0	2.0

^a)^The water soluble vitamin in mixture (in %): thiamine, 0.5; riboflavin, 0.5; pyridoxine, 0.5; calcium pantothenate, 2.8; nicotinamide, 2.0; inositol, 20.0; B12, 0.002; foric acid, 0.2; vitamin biotin, 0.01; and glucose monohydrate, 3.7.

^c)^The Calcium (Ca) Phosphorus (P) free salt mixture (in %): potassium chloride,57.7; sodium chloride,20.9;magnesium sulfate,anhydrous,17.9; copper(II)sulfate pentahydrate,0.078;sodium fluoride,0.113;cobalt(II)chloride,0.004;potassium lodide,0.01; magnese(II)sulfate pentahydrate,0.06; hexaammonium heptamolybdate tetrahydrate,0.005; iron(II)sulfate heptahydrate,3.22;zinc sulfate heptahydrate,0.44.

### Exercise

Exercise group rats were trained 6 days per week on a treadmill (KN-73, Natsume, Tokyo). The running speed and time were gradually increased (10–25 m/min, 10–60 min). Regular training started on the second week, and the running speed was further increased (25–30 m/min). Finally, the rats ran for 60 consecutive minutes (27–30 m/min). The training period was 60 days. This running speed (30 m/min) corresponds to 60 ~ 70% VO_2_max for rats [17]. To this training was added a warm-up session (15 m/min, 5 min) and a cool-down session (15 m/min, 5 min), making the total exercise time to 70 minutes.

### Dissection

After 11 weeks (at 16 weeks of age), rats were fasted for 12 h and dissected. The femur, tibia and lumbar spine were collected, and cleaned of adjacent tissues. The length of femora was immediately measured, and stored at 4°C for later mechanical testing. The tibiae and lumbar spines were stored in 70% ethanol for bone mineral content assessment.

### Bone mineral content

The BMC and area of lumbar spines and tibiae were analyzed by dual-energy X-ray absorptiometry (DXA: Aloka DCS-600R) [18].

### Femoral weights, length and mechanical testing

Femoral length was measured with a precision caliper. The femoral breaking force and energy were measured by the three point bending method using a bone strength measuring apparatus (Iio Co., Japan) as described in a previous report [19]. Subsequently, the femora were dried at 100°C for 24 h in the electric furnace, and their dry weight were measured. Next, the dried femur were burned to ash at 600°C for 15 h, and their ash weight were measured. The data of femoral breaking force and energy were adjusted to the dry weight (the adjusted breaking force and energy) to exclude the influence of body mass.

### Bone metabolic marker

Serum bone-specific alkaline phosphatase (BAP) activity, the bone mineralization parameters and tartrate-resistant acid phosphatase (TRAP) activity, and the bone resorption markers were determined as previously reported [20].

### Statistical methods

The results are expressed as the mean ± standard error of the mean (SE) and were analyzed with SPSS (version 21.0 J; SPSS Inc., Chicago, IL, USA). The data were analyzed using a two-way analysis of variance (ANOVA). Moreover, *t*-test was performed on four pairs of 20% protein groups and 40% protein groups of the same diet and physical activity to assess significant difference between the moderate and the higher protein groups (Casein20 × Casein40, Casein20 + Ex × Casein40 + Ex, HC20 × HC40, HC20 + Ex × HC40 + Ex). Statistical significance was taken at the p < 0.05 level.

## Results

### Food intake and body weight

At the beginning of the experiment, body weight did not differ among the groups. In the food intake during experiment, exercise effect was obtained (p < 0.001), and was significantly lower in the exercise groups than in the sedentary groups. These effects were detected both among the 20% protein groups and the 40% protein groups (Table 2). Therefore, the body weight gain, the food efficiency, and the final body weight were significantly lower in the exercise groups than in the sedentary groups (p < 0.001, respectively). Dietary HC effect was not obtained in these data among the 20% protein groups, but the effect was obtained in the food intake, the body weight gain, the food efficiency, and the final body weight among the 40% protein groups (p < 0.05, p < 0.01, p < 0.05 and p < 0.05, respectively, casein groups > HC groups) (Table 2). The food intake was significantly higher in the Casein20, HC20, and HC20 + Ex groups than the Casein40 (p < 0.01), HC40 (p < 0.01) and HC40 + Ex groups (p < 0.05, respectively) (Table 2).

**Table 2:** Body weight, body weight gain, food intake, energy intake, and food efficiency

		**20% protein**	**Two-way ANOVA (p value)**			**40% protein**	**Two-way ANOVA (p value)**		
			**Exercise**	**Collagen**	**Interaction**		**Exercise**	**Collagen**	**Interaction**
Initial body weight (g)									
Collagen(-)	EX(-)	115.3 ± 0.9	0.739	0.665	0.787	113.7 ± 2.1	0.759	0.218	0.240
	EX(+)	116.1 ± 1.5				115.5 ± 0.7			
Collagen(+)	EX(-)	116.3 ± 1.6				116.6 ± 1.2			
	EX(+)	116.4 ± 1.8				115.6 ± 0.6			
Final body weight (g)										
Collagen(-)	EX(-)	405.3 ± 5.5	<0.001	0.250	0.350	411.7 ± 8.8	<0.001	0.014	0.903	
	EX(+)	342.0 ± 7.2				354.1 ± 8.5				
Collagen(+)	EX(-)	391.0 ± 8.5				391.5 ± 5.4				
	EX(+)	340.5 ± 7.3				335.7 ± 8.7				
Body weight gain (g/d)										
Collagen(-)	EX(-)	4.0 ± 0.1	<0.001	0.189	0.259	4.1 ± 0.1	<0.001	0.006	0.758	
	EX(+)	3.1 ± 0.1				3.2 ± 0.1				
Collagen(+)	EX(-)	3.7 ± 0.1				3.7 ± 0.1				
	EX(+)	3.0 ± 0.1				3.0 ± 0.1				
Food intake (g/d)										
Collagen(-)	EX(-)	20.9 ± 0.2	<0.001	0.215	0.147	19.9 ± 0.2**	<0.001	0.019	0.712	
	EX(+)	18.2 ± 0.4				17.9 ± 0.3				
Collagen(+)	EX(-)	20.2 ± 0.2				19.3 ± 0.2**				
	EX(+)	18.3 ± 0.3				17.5 ± 0.12*				
Food efficiency^1^										
Collagen(-)	EX(-)	0.19 ± 0.00	<0.001	0.224	0.784	0.20 ± 0.00**	<0.001	0.028	0.926	
	EX(+)	0.17 ± 0.00				0.18 ± 0.00*				
Collagen(+)	EX(-)	0.18 ± 0.01				0.19 ± 0.00				
	EX(+)	0.16 ± 0.00				0.17 ± 0.01				

^1^Food efficiency was calculated by Body weight gain (g/d)/Food intake (g/d). EX(−): sedentary group, EX(+): exercise group.

Values are expressed as means ± SE. Data were analyzed by two-way ANOVA at the 5% level of significance. Interaction means Exercise-Collagen interaction. *p < 0.05 and *p < 0.01 vs. respective 20% protein group.

### BMC

**Figure 1:**
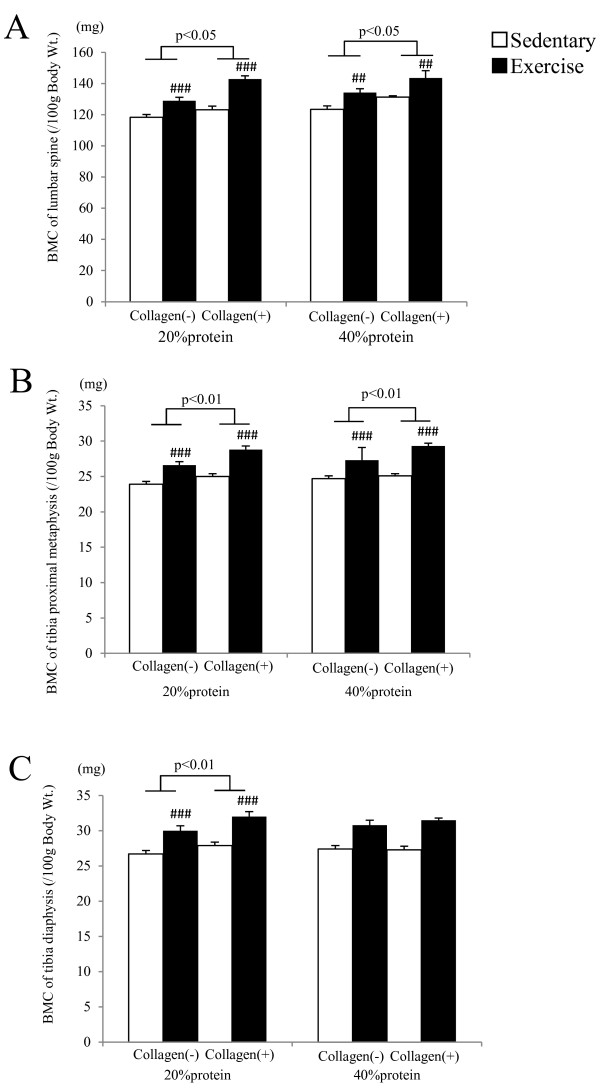
**Adjusted bone mineral content of lumbar spine, tibia proximal metaphysis, and tibia diaphysis.** Bone mineral content of lumbar spine **(A)**, tibia proximal metaphysis **(B)** and tibia diaphysis **(C)** adjusted to the 100 g body weight. The lumbar spine and tibia of each rat were isolated by dissection, and muscle and connective tissue were carefully removed. BMC was then measured by dual-energy X-ray absorptiometry. Vertical bars indicate the standard error. p value indicates statistical significant difference among dietary protein groups.

Exercise and dietary HC effects were obtained in the adjusted BMC of lumbar spine, tibia proximal metaphysis, and tibia diaphysis among the 20% protein groups (p < 0.001 for exercise, p < 0.05 for dietary HC, respectively). These adjusted BMC values were significantly higher in the exercise groups than those in the sedentary groups, and were also significantly higher in the HC groups than those in the casein groups. Among the 40% protein groups, similar results were obtained except for tibia diaphysis (p < 0.01 for exercise; p < 0.05 for dietary HC, respectively) (Figure 1). There were no differences between the 20% protein groups and the 40% protein groups.

### Femoral weights and length

Exercise and dietary HC effects were obtained in the adjusted wet weight and dry weight of femur among the 20% protein groups (p < 0.001, p < 0.01 for exercise; p < 0.01, p < 0.001 for dietary HC, respectively). In the adjusted ash weight, exercise effect was obtained among the 20% protein groups (p < 0.001). Among the 40% protein groups, similar results were obtained for exercise (p < 0.001, respectively) and for dietary HC (p < 0.01, p < 0.001, p < 0.01, respectively) (Table 3). There were no differences between the 20% protein groups and the 40% protein groups. In femoral length, the exercise groups were lower than the sedentary groups in both the 20% protein groups and the 40% protein groups, and exercise effect was obtained among the 20% protein groups (p < 0.01) (Table 3). Dietary HC effect was not obtained in femoral length both among the 20% protein groups and the 40% protein groups.

**Table 3:** Femoral weights and length

		**20% protein**	**Two-way ANOVA (p value)**			**40% protein**	**Two-way ANOVA (p value)**		
			**Exercise**	**Collagen**	**Interaction**		**Exercise**	**Collagen**	**Interaction**
Wet weight (g)									
Collagen(-)	EX(-)	0.9860 ± 0.0010	0.189	0.116	0.888	1.0127 ± 0.0206	0.326	0.570	0.271
	EX(+)	0.9633 ± 0.0290				0.9712 ± 0.0107			
Collagen(+)	EX(-)	1.0191 ± 0.0215				1.0020 ± 0.0159			
	EX(+)	0.9910 ± 0.0145				1.0044 ± 0.0319			
Wet weight (g/100g Body Wt.)									
Collagen(-)	EX(-)	0.2434 ± 0.0026	<0.001	0.006	0.633	0.2461 ± 0.0045	<0.001	0.001	0.191
	EX(+)	0.2796 ± 0.0077				0.2772 ± 0.0037			
Collagen(+)	EX(-)	0.2605 ± 0.0032				0.2560 ± 0.0035			
	EX(+)	0.2918 ± 0.0057				0.2988 ± 0.0066			
Dry weight (g)									
Collagen(-)	EX(-)	0.6363 ± 0.0088	0.013	0.152	0.540	0.6401 ± 0.0126	0.327	0.207	0.508
	EX(+)	0.6031 ± 0.0110				0.6202 ± 0.0075			
Collagen(+)	EX(-)	0.6450 ± 0.0142				0.6475 ± 0.0082			
	EX(+)	0.6247 ± 0.0088				0.6436 ± 0.0199			
Dry weight (g/100g Body Wt.)									
Collagen(-)	EX(-)	0.1570 ± 0.0021	0.001	<0.001	0.851	0.1556 ± 0.0028	<0.001	<0.001	0.365
	EX(+)	0.1751 ± 0.0027				0.1769 ± 0.0021			
Collagen(+)	EX(-)	0.1649 ± 0.0021				0.1654 ± 0.0016			
	EX(+)	0.1838 ± 0.0028				0.1915 ± 0.0040			
Ash weight (g)									
Collagen(-)	EX(-)	0.3981 ± 0.0109	0.193	0.572	0.686	0.4040 ± 0.0125	0.726	0.442	0.751
	EX(+)	0.3793 ± 0.0117				0.3972 ± 0.0037			
Collagen(+)	EX(-)	0.3998 ± 0.0128				0.4086 ± 0.0071			
	EX(+)	0.3899 ± 0.0108				0.4083 ± 0.0175			
Ash weight (g/100g Body Wt.)									
Collagen(-)	EX(-)	0.0982 ± 0.0016	<0.001	0.095	0.896	0.0982 ± 0.0027	<0.001	0.005	0.688
	EX(+)	0.1101 ± 0.0026				0.1134 ± 0.0024			
Collagen(+)	EX(-)	0.1022 ± 0.0016				0.1044 ± 0.0012			
	EX(+)	0.1147 ± 0.0034				0.1215 ± 0.0034			
Ash weight (g/Dry weight)									
Collagen(-)	EX(-)	0.6252 ± 0.0069	0.553	0.396	0.985	0.6310 ± 0.0033	0.223	0.577	0.540
	EX(+)	0.6287 ± 0.0042				0.6413 ± 0.0094			
Collagen(+)	EX(-)	0.6200 ± 0.0044				0.6313 ± 0.0038			
	EX(+)	0.6237 ± 0.0083				0.6347 ± 0.0037			
Length (cm)									
Collagen(-)	EX(-)	3.710 ± 0.014	0.004	0.216	0.109	3.696 ± 0.015	0.084	0.851	0.082
	EX(+)	3.623 ± 0.023				3.646 ± 0.009			
Collagen(+)	EX(-)	3.699 ± 0.017				3.668 ± 0.010			
	EX(+)	3.675 ± 0.018				3.669 ± 0.023			
Long Width (cm)									
Collagen(-)	EX(-)	0.440 ± 0.005	0.848	0.266	0.722	0.441 ± 0.005	1.000	0.035	0.339l
	EX(+)	0.438 ± 0.004				0.436 ± 0.003			
Collagen(+)	EX(-)	0.444 ± 0.006				0.446 ± 0.005			
	EX(+)	0.445 ± 0.005				0.451 ± 0.006			
Short Width (cm)									
Collagen(-)	EX(-)	0.352 ± 0.004	0.169	0.328	0.591	0.348 ± 0.005	0.121	0.385	0.746
	EX(+)	0.345 ± 0.003				0.344 ± 0.002			
Collagen(+)	EX(-)	0.346 ± 0.004				0.353 ± 0.003			
	EX(+)	0.343 ± 0.003				0.346 ± 0.005			

Values are expressed d as means ± SE. Data were analyzed by two-way ANOVA at the 5% level of significance. Interaction means Exercise-Collagen interaction.

### Bone breaking force and energy of femur

Among the 20% protein groups, exercise effect was obtained in the adjusted femoral breaking force and energy (p < 0.01, respectively) and the exercise groups were significantly higher than those in the sedentary groups, whereas dietary HC effect was not significant (Table 4). Similarly, among the 40% protein groups, exercise effect was only obtained in the adjusted femoral breaking force and energy (p < 0.01 for adjusted breaking force; p < 0.05 for adjusted breaking energy), and dietary HC effect was not significant (Table 4). There were no differences in both the adjusted femoral breaking force and energy between the 20% protein groups and the 40% protein groups.

**Table 4:** Breaking force and energy of the femoral diaphysis

		**20% protein**	**Two-way ANOVA (p value)**			**40% protein**	**Two-way ANOVA (p value)**		
			**Exercise**	**Collagen**	**Interaction**		**Exercise**	**Collagen**	**Interaction**
Breaking force (×10^6^ dyn)									
Collagen(-)	EX(-)	29.358 ± 1.396	0.574	0.523	0.068	27.864 ± 1.105	0.757	0.708	0.547
	EX(+)	26.702 ± 0.928				29.132 ± 1.994			
Collagen(+)	EX(-)	26.618 ± 1.358				29.222 ± 1.101			
	EX(+)	28.037 ± 0.803				28.816 ± 1.255			
Breaking force (×10^6^ dyn/100g Body Wt.)^1^									
Collagen(-)	EX(-)	7.234 ± 0.329	0.001	0.909	0.082	6.766 ± 0.227	0.002	0.274	0.605
	EX(+)	7.741 ± 0.231				8.343 ± 0.179			
Collagen(+)	EX(-)	6.798 ± 0.31				7.455 ± 0.254			
	EX(+)	8.237 ± 0.218				8.591 ± 0.352			
Breaking energy (×10^5^ erg)									
Collagen(-)	EX(-)	20.301 ± 1.598	0.458	0.919	0.182	17.202 ± 1.778	0.492	0.195	0.145
	EX(+)	19.430 ± 1.116				20.546 ± 1.048			
Collagen(+)	EX(-)	18.203 ± 1.704				21.499 ± 1.280			
	EX(+)	21.231 ± 1.480				20.290 ± 1.982			
Breaking energy (×10^5^ erg/100d Body Wt.)^1^									
Collagen(-)	EX(-)	4.987 ± 0.37	0.002	0.886	0.269	4.191 ± 0.436	0.010	0.070	0.190
	EX(+)	5.758 ± 0.221				5.833 ± 0.296			
Collagen(+)	EX(-)	4.644 ± 0.407				5.496 ± 0.376			
	EX(+)	6.202 ± 0.389				6.047 ± 0.569			

Values are expressed as means ± SD. Data were analyzed by two-way ANOVA at the 5% level of significance. Interaction means Exercise-Collagen interaction.

^1^Breaking force and energy adjusted to the 100g body weight to exclude the influence of body mass.

### Bone metabolic marker

BAP activity did not differ among the 20% protein groups (Casein20: 38.70 ± 15.20U/L, Casein20 + Ex: 55.28 ± 12.14U/L, HC20: 33.91 ± 8.91U/L, HC20 + Ex: 33.91 ± 10.16U/l). Similarly, among the 40% protein groups, there were no differences (Casein40: 35.75 ± 8.69U/l, Casein40 + Ex: 38.14 ± 10.01U/l, HC40: 33.31 ± 7.90U/l, HC40 + Ex: 37.66 ± 7.58U/l). Moreover, TRAP activity did not also differ among the 20% and 40% protein groups, respectively (Casein20: 19.39 ± 2.11U/L, Casein20 + Ex: 24.59 ± 3.36U/L, HC20: 17.75 ± 3.97U/L, HC20 + Ex: 18.81 ± 2.20U/L, Casein40: 19.65 ± 1.27U/L, Casein40 + Ex: 22.10 ± 4.47U/L, HC40: 20.47 ± 1.43U/L, HC40 + Ex: 21.75 ± 1.67U/L). In these two bone metabolic markers, there were no differences between the 20% protein groups and the 40% protein groups.

## Discussion

We investigated the effects of HC intake and treadmill running exercise on bone mass and strength in growing male rats. This study demonstrated that HC intake increases bone mass in both trained and untrained growing rats. Although these results were shown in both moderate and high protein intake groups, the level of these beneficial effects on bone mass was similar for the two groups. The intake of a high protein diet containing HC may have no more beneficial effect on bone mass and strength on growing rats trained with running exercise than the intake of a moderate protein diet containing HC.

In the present study, we showed the effect of HC intake and treadmill running exercise on adjusted BMC of lumbar spine and tibia. The adjusted BMC was higher in the exercise groups (Casein20 + Ex, Casein40 + Ex, HC20 + Ex, and HC40 + Ex) than in the sedentary groups (Casein20, Casein40, HC20, and HC40). Especially in the trained HC intake groups (HC20 + Ex, HC40 + Ex), those effects were strongly observed. Guillerminet et al. [21] had shown that the BMD for OVX mice fed with the diet including HC (porcine origin) was significantly higher as compared to OVX mice fed on a standard AIN-93N diet. Mizoguchi et al. [22] had also shown that the HC (marine fish origin) intake increased the level of serum osteocalcin (OC), a well-known marker of osteogenesis, along with the BMD and the bone strength of femur in OVX rats. The levels of serum hydroxyproline and glycine of the HC intake group were increased in those cases. These results suggest that dietary HC intake increases the level of serum amino acid (hydroxyproline and glycine), the important components of bone, which then increases the BMD and bone strength. Moreover, *in vitro* study, hydrolyzed collagens (bovine, porcine, and fish origin, respectively having a molecular weight of 2 or 5 kDa) in osteoblasts had significant and dose-dependent increase in ALP activity, a well-known marker of osteogenesis [23]. These results suggest that dietary hydrolyzed collagen may increase bone formation. Although, our result did not show the difference of bone formation marker, we cautiously postulated that the beneficial effect of HC intake in this study could have acted on bone during growth phase since we assessed the bone markers by end-point experiment when being already adult bone. Taken together, these results suggest that HC intake has a beneficial effect on bone mass in growing rats and this effect is more beneficial for rats participating in treadmill running exercise.

Our study also investigated whether the intake of a high protein diet containing HC has positive effects on bone mass and strength of growing rats trained with running exercise. Although the adjusted BMC of lumbar spine and tibia were significantly higher in the HC groups than in the casein groups among both the 20% protein groups and the 40% protein groups, the dietary HC effect had no dose-dependent increase. In the previous study, we had shown that a high protein, two times the 20% moderate casein intake, had no positive effect on bone mass and strength in growing rats [14]. Moreover, in this study, the intake of a high protein diet containing HC also had no more beneficial effect than a moderate protein diet containing HC on bone mass and strength in growing rats. These results suggest that the beneficial effect of HC intake on increasing bone mass may have been limited.

Interestingly, the beneficial effect of HC intake was not observed on bone strength. Seventy percent of bone strength depends on its density and 30% depends on its quality [24]. The bone quality is determined by the degree of bone mineralization, microdamage accumulation, bone size, collagen crosslinks formation and bone turnover rate [25]. Thus, the reason for the same level of bone strength between the casein groups and the HC intake groups despite the higher level of bone mass in the HC intake groups than in the casein intake groups might be in the bone quality difference. Mizoguchi et al. had investigated that mineral and collagen derived from fish-skin supplementation tend to improve bone strength in OVX rats [26]. On the other hand, there are very few studies investigating the effect of HC intake on bone strength during growth phase. Our data suggest that the effect of HC intake may change in different bone statuses. More investigation is necessary to discuss the effect of HC intake on bone quality and strength.

Our study had several limitations. The food intake and final body weight were significantly lower in the exercise groups than in the sedentary groups. Moreover, among the 40% protein groups, these data were significantly lower in the HC intake groups than in the casein intake groups. Growth of bone is considerably influenced by body mass [27]. Therefore, we were unable to precisely describe the relation of weight-related growth and benefits of physical exercise. Moreover, we had investigated the effect of HC intake by exchanging a part of casein with HC (30% of protein was HC). Therefore, the amount of carbohydrate and essential amino acids were different between 20% protein diet and 40% protein diet. This may have had some effect on the results of our previous study. Further research is needed to assess the effect of HC intake in conditions where the amount of other nutrition is adjusted.

In summary, the present study demonstrated that moderate HC intake (where the diet contains 20% protein, of which 30% is HC) increased bone mass during growth periods and further promoted the effect of running exercise. On the other hand, a high protein diet containing HC (where the diet contains 40% protein, of which 30% is HC) had no more beneficial effect on bone mass than the moderate protein intake. These results could be of potential interest for new nutritional support in the increasing effect of physical exercise on bone mass during growth.

## Competing interests

The authors declare no competing interests.

## Authors’ contributions

ST conceived of the study and carried out: 1) study design, 2) data collection, 3) data analysis, 4) statistical analysis and 5) preparing manuscript. JHP assisted in 1) data analysis and 2) preparing the manuscript. EK assisted in 1) study design and 2) data collection. IE assisted in coordination and helped to draft the manuscript. NO procured grant funding and assisted in: 1) study design, 2) data collection and analysis, and 3) preparing the manuscript. All authors read and approved the final manuscript.
